# Intraperitoneal Chemotherapy Using Fluorouracil Implants Combined With Radical Resection and Postoperative Adjuvant Chemotherapy for Stage III Gastric Cancer: A Multi-Center, Randomized, Open-Label, Controlled Clinical Study

**DOI:** 10.3389/fonc.2021.670651

**Published:** 2021-07-08

**Authors:** Yan Xu, Rupeng Zhang, Chunfeng Li, Zhe Sun, Jingyu Deng, Xiaona Wang, Xuewei Ding, Baogui Wang, Qiang Xue, Bin Ke, Hongjie Zhan, Ning Liu, Yong Liu, Xuejun Wang, Han Liang, Yingwei Xue, Huimian Xu

**Affiliations:** ^1^ Department of Surgical Oncology and General Surgery, The First Hospital of China Medical University, Shenyang, China; ^2^ Department of Gastric Cancer, Tianjin Medical University Cancer Institute & Hospital, National Clinical Research Center of Cancer, Key Laboratory of Cancer Prevention and Therapy, Tianjin’s Clinical Research Cancer for Cancer, Tianjin, China; ^3^ Department of Gastroenterological Surgery, Harbin Medical University Cancer Hospital, Harbin, China

**Keywords:** gastric cancer, intraperitoneal chemotherapy, fluorouracil implants, metastasis, survival

## Abstract

**Background:**

Reducing peritoneal recurrence after radical surgery is an important choice to improve the prognosis of patients with advanced gastric cancer. Intraoperative intraperitoneal chemotherapy has the potential to be a promising treatment strategy. In the present study, we conducted a multi-center, randomized, controlled clinical study to evaluate the efficacy and safety of intraoperative intraperitoneal chemotherapy using sustained-release fluorouracil implants plus radical gastrectomy and adjuvant chemotherapy for cTNM stage III gastric cancer.

**Methods:**

The patients were randomized into intraperitoneal chemotherapy group (sustained-release fluorouracil implants administration after standard D2 radical gastrectomy, and followed by XELOX adjuvant chemotherapy) and control group (standard D2 radical gastrectomy, and followed by XELOX adjuvant chemotherapy). A total of 122 patients from three centers were enrolled from September 2015 to February 2017.

**Results:**

One hundred and two eligible patients completed the treatment course. The median follow-up time was 41.7 months (36.1–52.9 months). The 3-year progression-free survival rate and overall survival of patients in the intraperitoneal chemotherapy group were 43.9% and 49.1%, respectively, which were significantly better than those of the control group, 31.0% and 38.4%. In the intraperitoneal chemotherapy group, the number of cases with peritoneal recurrence was significantly less than that of the control group, 9 cases (17.3%) *vs.* 19 cases (44.2%). There were neither significant differences between the groups in the incidence of hematogenous metastasis, lymph node metastasis, nor local metastasis.

**Conclusion:**

For cTNM stage III gastric cancer, intraoperative sustained-release fluorouracil implants after radical resection combined with postoperative adjuvant chemotherapy, could significantly reduce the risk of peritoneal recurrence and prolong PFS.

**Clinical Trial Registration**

https://clinicaltrials.gov/, identifier (NCT02269904).

## Introduction

Gastric cancer is one of the most common malignancies throughout the world and also one of the leading causes of cancer-related deaths. Recurrence after gastrectomy, especially peritoneal recurrence, is a primary factor affecting the survival of gastric cancer patients. Lee et al. (1) showed that recurrence affected the abdominal cavity in over half of the gastric cancer patients (58.8%). Moreover, the estimated median survival time after peritoneal recurrence was only 9.4 months. The risk factors closely associated with peritoneal recurrence include tumor invasion of serosa (>T3), lymph node metastases (N3), and Borrmann type 4 gastric cancer (2). The causes for peritoneal recurrence include the presence of free cancer cells before surgery (CY+), release of cancer cells from the severed lymphatic vessels, and residual blood contaminated by tumor cells in the peritoneal cavity (3).

Conventional intravenous chemotherapy (systemic administration) can hardly achieve the effective drug concentration in the peritoneal cavity because of the peritoneal-plasma barrier. Besides, intravenous chemotherapy only has a limited killing effect on the intra-abdominal cancer cells due to deficiency of vasculature and hypoxia in peritoneal tumor (4). In contrast, intraperitoneal chemotherapy can achieve a higher local drug concentration without severe systemic reaction. A growing number of studies have shown that intraoperative or postoperative intraperitoneal chemotherapy can dramatically reduce the risk of postoperative peritoneal recurrence and increase the survival rate (5, 6). In recent years, intraoperative prophylactic intraperitoneal chemotherapy gradually becomes applied in clinical practice (7, 8). The randomized controlled trial using hyperthermic intraperitoneal chemotherapy (HIPEC) in patients at risk for peritoneal metastases of gastric cancer was reported by Yonemura et al. (9). The results showed that as compared with simple radical resection, radical resection combined with HIPEC could significantly improve the 5-year overall survival (61% for HIPEC+surgery *vs.* 42% for surgery).

In addition to HIPEC, there are some user-friendly, fewer side effect treatments that are also evaluated in intraperitoneal chemotherapy, such as catheter-based and sustained-release drug implants intraperitoneal chemotherapy. Catheter-based intraperitoneal chemotherapy is allowed for repeated administration through a retained peritoneal catheter and is much less invasive compared with HIPEC (10). A retrospective study showed that catheter-based early postoperative intraperitoneal chemotherapy (EPIC) as a prophylactic treatment for gastric cancer with serosal invasion significantly decreased the peritoneal recurrence and improved survival time (11).

In recent years, sustained-release fluorouracil implants intraoperative intraperitoneal chemotherapy is gaining attention. Because implants or microspheres for sustained-release drug delivery are given only once during surgery, and are long-acting, they have drawn increasing attention. Yuan et al. applied intraoperative intraperitoneal chemotherapy using sustained-release fluorouracil implants to advanced colorectal cancer. As compared with the simple surgery group, the intraoperative chemotherapy group using sustained-release fluorouracil implants showed a significant reduction in the local recurrence rate and an improved PFS (12).

Here, a multi-center, randomized, open-label, controlled clinical study was conducted to observe the efficacy and safety of intraoperative intraperitoneal chemotherapy using sustained-release fluorouracil implants combined with radical gastrectomy and adjuvant chemotherapy for stage III gastric cancer.

## Materials and Methods

### Participants

A multi-center, randomized, open-label, controlled clinical study was conducted to evaluate the efficacy and safety of intraoperative intraperitoneal chemotherapy using fluorouracil implants plus radical gastrectomy and adjuvant chemotherapy for stage III gastric cancer. Inclusion criteria: pathologically confirmed gastric adenocarcinoma, serosa invasion and lymph node metastases indicated by preoperative CT scan and intraoperative examination, having received radical gastrectomy (D2) to achieve R0 resection, aged 18 or above, KPS≥70%. Exclusion criteria: having received chemotherapy, radiotherapy, or immunotherapy before surgery; distant metastases (liver, ovaries, and omentum) found intraoperatively or confirmed by postoperative pathological examination; positive peritoneal cytology; having definite diseases or abnormal laboratory test results, or other conditions that made the researchers determine that the subjects were not fit for the study.

### Experimental Design

Participants were recruited from the gastric cancer patients who were treated at three centers from September 2015 to February 2017, namely First Affiliated Hospital of China Medical University, Tianjin Medical University Cancer Institute and Hospital, and Heilongjiang Province Cancer Hospital. Patients who met the study criteria were randomly assigned to the intraperitoneal chemotherapy group and control group at a 1:1 scale. The randomization sequence was generated independently by computer using random permuted blocks, stratified by histological differentiation type (differentiated *vs.* undifferentiated) and ECOG score (0 *vs.* 1, 2). This is an open study.

Patients in the control group only received standard D2 surgical resection and postoperative adjuvant chemotherapy using the oxaliplatin/capecitabine (XELOX) regimen. For the intraperitoneal chemotherapy group, fluorouracil implants were placed intraoperatively (Sinofuan^®^, Jiangsu Simcere Pharmaceutical Co., Ltd./Wuhu Simcere Sino-implant Pharmaceutical Co., LTD), after standard D2 radical gastrectomy, and followed by XELOX adjuvant chemotherapy.

The protocol was approved by the ethics committees of the three centers (the medical ethics committee of the First Hospital of China Medical University, the ethics committee of Tianjin Medical University Cancer Institute & Hospital, and the ethics committee of Harbin Medical University Cancer Hospital). The present study was registered at clinicaltrials.gov (https://clinicaltrials.gov/) on October 21, 2014, and the registration number was NCT02269904. The study conformed to the Declaration of Helsinki. Every participant signed the informed consent before recruitment.

### Experimental Workflow

All participants were of stage III gastric cancer according to preoperative evaluation and were interviewed before recruitment. If the participants consented to the study, they signed an informed consent. The peritoneal cavity was examined first during surgery, and the lesions were evaluated. If the patients conformed to the inclusion criteria, they were randomized to different groups. Radical gastrectomy combined with D2 lymphadenectomy was performed, depending on the lesions. All surgeries were performed by an experienced surgical oncologist who was specialized in gastric cancer. Before closing the abdomen, fluorouracil implants were placed scatteredly into the peritoneal cavity at a total dose of 800 mg. The fluorouracil implants were mainly placed in the tumor bed, pelvic cavity, paracolic sulci, and subdiaphragm, which were avoided on the surface of the small intestine, in the anastomotic stoma, or the exposed blood vessels.

### Evaluation

Primary endpoints: the 3-year progression-free survival (PFS) was evaluated in the two groups. Secondary endpoints: ① peritoneal metastasis rate; ② overall survival (OS); ③ safety of the fluorouracil implants.

Safety evaluation: within 1 month after surgery, all adverse events and complications were recorded. Throughout the follow-up period of the study, if an adverse event is shown to be related to it, it is also recorded.

Follow-up: all patients were follow-up and received regular examinations, including physical examination, tumor marker test, abdominal, and pelvic CT. These examinations were performed once every three months during the first 2 months after surgery. Since the third year after surgery, they were performed once every 6 months; since the fifth year after surgery, they were performed once every year.

Diagnostic criteria for peritoneal recurrence: ① unevenly thickened or enhanced nodules in the peritoneum or omentum found by CT scan, with increased levels of tumor marker; ② massive pelvic effusion, with cancer cells found by cytology; ③ peritoneal metastasis found by another surgery due to intestinal obstruction and pathologically confirmed.

Hematogenous recurrence included liver metastasis, lung metastasis, and osseous metastasis. Lymphatic recurrence covered Virchow’s node metastasis, para-aortic lymph node metastasis, and retroperitoneal lymph node metastasis. Locoregional recurrence included recurrence in the remnant stomach, stomach bed, and bile duct.

### Statistics

The Graphpad Prism 8 software was used for statistical analysis. Quantitative indicators were described by means, standard deviation, median, maximum, and minimum. Intergroup comparison was conducted by using the log-rank test. Count indicators were described by number and percentages; the intragroup comparison was performed by using the chi-square test. All statistical tests were two-sided. P<0.05 indicated a significant difference.

## Results

### Baseline Information of the Participants

**Figure 1:**
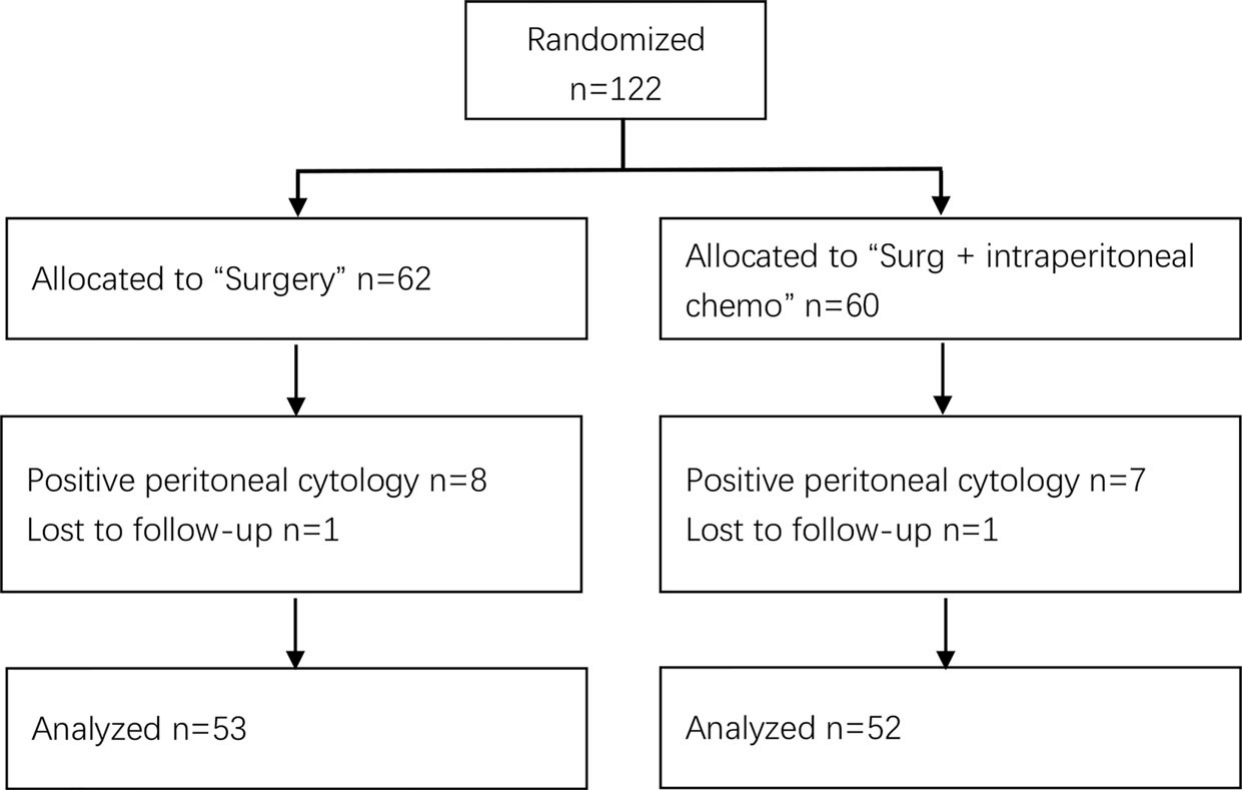
Flowchart of the participants.

From September 2015 to February 2017, a total of 122 participants were recruited from the three centers. They were randomly divided into intraperitoneal chemotherapy group (n=60) and control group (n=62). In the intraperitoneal chemotherapy group, seven cases were excluded (CY+), with one dropout, and 52 cases were left; in the control group, eight cases were excluded (CY+), with one dropout, and 53 cases were left. The flowchart of the study is shown in **Figure 1**. The median follow-up time was 41.7 months (36.1–52.9 months). The average age of the patients in the intraperitoneal chemotherapy group was 59.3 years (23–75 years), and that of the control group was 62.6 years (25–88 years). The average body surface area of the patients in the intraperitoneal chemotherapy group was 1.78 m ^2^ (1.31–1.93 m^2^), and that of the control group was 1.72 m^2^ (1.20–1.82 m^2^). The average times of postoperative adjuvant chemotherapy in the intraperitoneal chemotherapy group was 7.34, compared to 6.95 in the control group. There was no significant difference between the two groups (P>0.05). The basic features of patients in the two groups were comparable statistically, and there were no significant differences, as shown in **Table 1**.

**Table 1:** Clinicopathological features of the included participants.

Characteristic	Intraperitoneal+adjuvant chemo (n=52)	Adjuvant chemo (n=53)	P value
Age, y (mean ± SD)	59.3 ± 1.374	62.6 ± 1.200	0.078
Gender, N (%)			
Male	34 (65.4%)	32 (60.4%)	0.687
Female	18 (34.6%)	21 (39.6%)	
Average body surface, m^2^ (mean ± SD)	1.78 ± 0.16	1.72 ± 0.15	0.089
Adjuvant chemotherapy time (mean ± SD)	7.34 ± 2.67	6.95 ± 2.64	0.425
KPS, (mean ± SD)	87.09 ± 8.74	89.81 ± 7.11	0.156
ECOG, N (%)			
0	12 (23.1%)	15 (28.3%)	0.237
1	39 (75.0%)	36 (67.9%)	
2	1 (1.9%)	2 (3.8%)	
3	0 (0)	0 (0)	
Histology			
Well differentiated	22 (42.3%)	25 (47.2%)	0.696
Poor differentiated	30 (57.7%)	28 (52.8%)	
Tumor location			
Upper	8 (15.4%)	11 (20.8%)	
Middle	10 (19.2%)	8 (15.1%)	
Lower	34 (65.4%)	34 (64.1%)	
Type of operation			
Total gastrectomy	17 (32.7%)	20 (37.7%)	0.684
Subtotal gastrectomy	35 (67.3%)	33 (62.3%)	
pTNM stage			
IIB	6 (11.5%)	5 (9.4%)	
IIIA	22 (42.3%)	21 (39.6%)	
IIIB	19 (36.5%)	21 (39.6%)	
IIIC	5 (9.6%)	6 (11.3%)	
pN stage			
0	2 (3.8%)	4 (7.5%)	
1	12 (23.1%)	13 (24.5%)	
2	12 (23.1%)	10 (18.9%)	
3	26 (50.0%)	26 (49.1%)	
3a	22 (42.3%)	18 (34.0%)	
3b	4 (7.7%)	8 (15.1%)	
pT stage			
2	2 (3.8%)	2 (3.8%)	
3	9 (17.3%)	8 (15.1%)	
4	41 (78.8%)	43 (81.1%)	
4a	40 (76.9%)	41 (77.4%)	
4b	1 (1.9%)	2 (3.8%)	

### Survival Analysis

**Figure 2:**
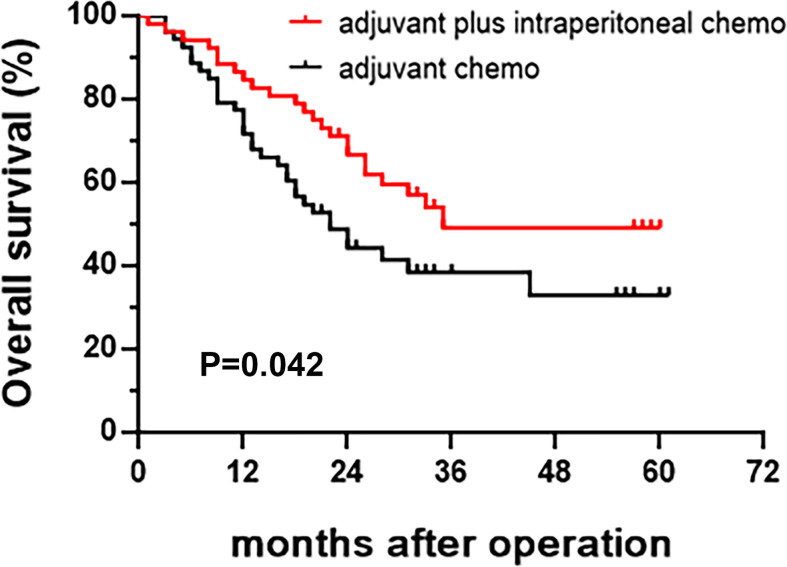
Kaplan-Meier curve of overall survival.

**Figure 3:**
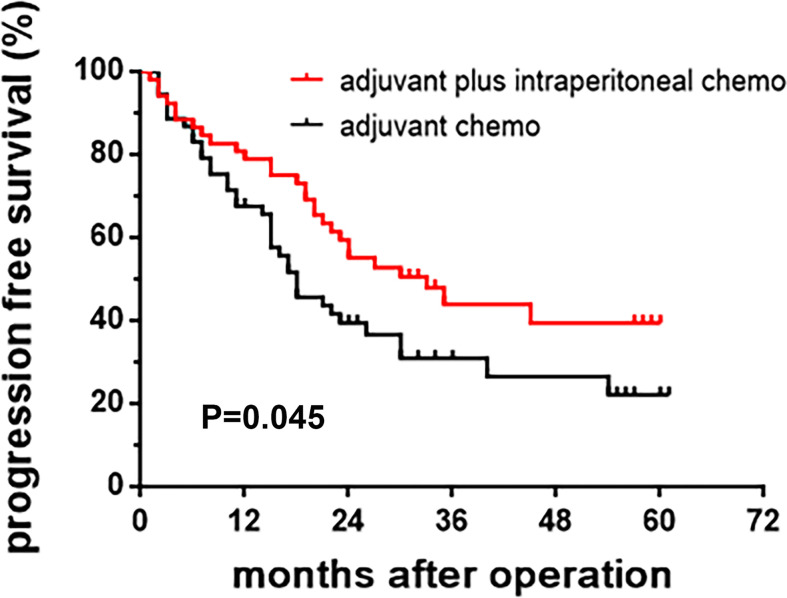
Kaplan-Meier curve of progression-free survival.

The 3-year PFS rate of patients in the intraperitoneal chemotherapy group was 43.9%, and that of the control group was 31.0%, significant differences between the two groups (P=0.045) (**Table 2**). The 3-year OS rate of patients in the intraperitoneal chemotherapy group was 49.1%, and that of the control group was 38.4%, significant differences between the two groups (P=0.042) (**Table 3**). The median survival time of patients in the intraperitoneal chemotherapy group was 35 months, and that of the control group was 21 months, without significant differences between the two groups. The survival curve is presented in **Figures 2** and **3**.

**Table 2:** 3-year PFS rate.

	n	3-year DFS	P value
Intraperitoneal chemotherapy group	52	43.9%	
Control group	53	31.0%	0.045

**Table 3:** 3-year OS rate.

	n	3-year OS	P value
Intraperitoneal chemotherapy group	52	49.1%	
Control group	53	38.4%	0.042

### Postoperative Complications

During the study period, there were no treatment-related deaths. There were no significant differences in the incidence of postoperative complications between the intraperitoneal chemotherapy group and the control group, such as anastomotic fistula, anastomotic bleeding, abdominal infection, and pulmonary infection. During the hospital stay, no intestinal obstruction or poor wound healing occurred. There were no significant differences in the gas passage time and drainage volume between the two groups. See **Tables 4** and **5**.

**Table 4:** Postoperative complications and surgical outcomes in the two groups.

	Intraperitoneal chemotherapy group (n=52)	Control group (n=53)	P
Anastomotic leakage	1	1	
Anastomotic bleeding	0	1	
Abdominal infection	2	1	
Ileus	1	0	
Pulmonary infection	1	3	
Gas passage time(days)	4.73 ± 1.17	4.94 ± 1.80	
Drainage volume(ml)			
1^st^ day	123.91 ± 83.07	121.34 ± 94.06	>0.05
2^nd^ day	88.55 ± 60.37	86.63 ± 71.32	>0.05
3^rd^ day	60.35 ± 60.00	63.47 ± 87.05	>0.05

**Table 5:** Toxic and adverse effects of the patients in the two groups.

	Intraperitoneal chemotherapy group (n=52)	Control group (n=53)
Surgical morbidity		
Anastomotic leakage	1 (1.9%)	1 (1.9%)
Bleeding	0	1 (1.9%)
Peritoneal abscess	2 (3.8%)	1 (1.9%)
Intestinal obstruction	0	0
Pancreatic fistula	2 (3.8%)	3 (5.7%)
Pneumonia	1 (1.9%)	3 (5.7%)
Non-surgical morbidity		
Creatinine >2.0	2 (3.8%)	1 (1.9%)
AST, ALT >100	7 (13.5%)	4 (7.5%)
Hospital death	0	0

### Recurrence

The postoperative recurrence rate of the intraperitoneal chemotherapy group was significantly lower than that of the control group (53.8% versus 67.9%). The recurrence at different sites was further compared between the two groups. The results showed that in the intraperitoneal chemotherapy group, there were nine cases of peritoneal recurrence (17.3%). The number of cases with peritoneal recurrence was significantly less than that of the control group, which was 19 (44.2%). There were neither significant differences in the incidence of hematogenous metastasis, lymph node metastasis, nor local metastasis (**Table 6**).

**Table 6:** Analysis of recurrence.

	Intraperitoneal chemotherapy group (n=52)	Control group (n=53)	P
No recurrence	24 (46.2%)	17 (32.1%)	
Recurrence	28 (53.8%)	36 (67.9%)	0.164
Peritoneal	9 (17.3%)	19 (44.2%)	0.006
Hematogenous	15 (28.8%)	16 (30.2%)	1.000
Loco-regional	11 (21.2%)	14 (26.4%)	0.648

## Discussion

For peritoneal recurrence from gastric cancer, cytoreductive surgery combined with hyperthermic intraperitoneal chemotherapy is proven to achieve remission and prolonged survival time (13, 14). On the other hand, for gastric cancer patients who have received radical surgery, reducing peritoneal recurrence after surgery is an important choice to improve the prognosis of the patients. An increasing number of studies have shown that postoperative/intraoperative intraperitoneal chemotherapy can significantly decrease the peritoneal recurrence rate and improve PFS in high-risk patients, such as those with stage III gastric cancer and with lymph nodes metastases (15–17). Postoperative/intraoperative intraperitoneal chemotherapy usually includes normothermic intraperitoneal chemotherapy, hyperthermic intraperitoneal chemotherapy, and intraoperative implants. One study has indicated that although early postoperative HIPEC could reduce the risk of peritoneal recurrence, the manipulations are tedious, and some special equipment is required. Moreover, the incidence of adverse events of grade III or IV remains high, which has restricted the application of HIPEC to adjuvant chemotherapy after radical surgery. Normothermic intraperitoneal chemotherapy is associated with a lower incidence of side effects when HIPEC as reference. However, because the chemotherapeutic drug is rapidly absorbed and degraded in the peritoneal cavity, the drug is short-acting and needs to be given repeatedly.

Sustained-release chemotherapeutic drugs allow for a slow release of the drug from the carrier, forming a high local concentration, which is long-acting and causes mild systemic side effects (18, 19). Therefore, they have been gradually applied to postoperative adjuvant chemotherapy for malignancies. It has been shown that intraoperative sustained-release fluorouracil implants combined with postoperative adjuvant chemotherapy following radical resection of ovarian cancer significantly reduced the risk of peritoneal recurrence and improved PFS. Besides, there was no significant difference in the incidence of side effects as compared with the control group (20). Intraperitoneal sustained-release implants have also been used to treat liver cancer (21) and colorectal cancer (22) with good outcomes. The present study was a multi-center, randomized, open-label, controlled clinical study that focused on the efficacy and safety of intraperitoneal chemotherapy using fluorouracil implants combined with radical resection and postoperative adjuvant chemotherapy for stage III gastric cancer. Our results suggested that for stage III gastric cancer, early intraperitoneal chemotherapy using sustained-release fluorouracil implants after radical resection combined with postoperative adjuvant chemotherapy could significantly reduce the risk of peritoneal recurrence and prolong PFS subsequently.

Fluorouracil is a classic chemotherapeutic drug which through inhibition of thymidylate synthase (TS) and incorporation of its metabolites into RNA and DNA achieves an anti-cancer effect (23). It is indispensable for many chemotherapy regimens in gastric cancer and intestinal cancer (24). However, the half-life of fluorouracil is short, and continuous intravenous injection or oral administration may be required for systemic chemotherapy based on fluorouracil (25, 26). In the present study, intraperitoneal fluorouracil implants, where fluorouracil was encapsulated within the microcapsules, were used. This formulation enabled sustained release of fluorouracil within the abdominal cavity. The drug release time lasted longer than 30 days (27). The problem of the short half-life of fluorouracil was thus well resolved.

Our results showed that intraoperative intraperitoneal fluorouracil implants did not increase the incidence of side effects, such as intestinal obstruction, fistula, and infection, as compared with the control group. Several existing studies on other tumors have demonstrated that intraperitoneal fluorouracil implants would not increase the side effects. Chen et al. placed fluorouracil implants on the resection margin of the liver, which only caused an increase in the drainage volume. There were no significant differences in liver function and incidence of bleeding and infection as compared with the control group (21). Another concern about late effect of fluorouracil implants is that the drug carrier was made of nonabsorbable material. There is a risk of abdominal adhesions and obstruction. In the present study, safety rules were followed to prevent side effects as much as possible. For example, fluorouracil implants were placed at high-risk sites, such as tumor bed, pelvic cavity, paracolic sulci, and subdiaphragm, and no implants were placed on the surface of the small intestine, anastomotic stoma, and exposed blood vessels.

It is concluded that for stage III gastric cancer, intraoperative sustained-release fluorouracil implants after radical resection combined with postoperative adjuvant chemotherapy could significantly reduce the risk of peritoneal recurrence and prolong PFS. As compared with the control group, the incidence of side effects was not increased significantly.

## Data Availability Statement

The original contributions presented in the study are included in the article/supplementary material. Further inquiries can be directed to the corresponding authors.

## Ethics Statement

The studies involving human participants were reviewed and approved by the medical ethics committee of the First Hospital of China Medical University. The patients/participants provided their written informed consent to participate in this study.

## Author Contributions

Trail design and development of the protocol: HX, HL, and YWX. Patients recruited and data collection: ZS, JD, XNW, XD, BW, QX, BK, HZ, NL, YL, and XJW. Drafting the manuscript: YX, RZ, CL, and ZS. All authors contributed to the article and approved the submitted version.

## Conflict of Interest

The authors declare that the research was conducted in the absence of any commercial or financial relationships that could be construed as a potential conflict of interest.
